# Cas9-targeted nanopore sequencing reveals epigenetic heterogeneity after *de novo* assembly of native full-length hepatitis B virus genomes

**DOI:** 10.1099/mgen.0.000507

**Published:** 2021-05-18

**Authors:** Chloe Goldsmith, Damien Cohen, Anaëlle Dubois, Maria Guadalupe Martinez, Kilian Petitjean, Anne Corlu, Barbara Testoni, Hector Hernandez-Vargas, Isabelle Chemin

**Affiliations:** ^1^​ INSERM U1052, CNRS UMR-5286, TGFB and Immune Evasion, Lyon Cancer Research Center (CRCL), Lyon, France; ^2^​ INSERM U1052, CNRS UMR-5286, Cancer Research Center of Lyon (CRCL), Lyon, France; ^3^​ Inserm, Univ-Rennes, INRAe, Institut Nutrition, Metabolism and Cancer (NuMeCan), UMR_S 1241, Rennes, France; ^4^​ Centre Leon Berard (CLB), Lyon Cancer Research Center (CRCL), Lyon, France

**Keywords:** DNA methylation, nanopore, epigenetics, 5mC, HBV, ONT, cccDNA, cirrhosis, HCC, long-read sequencing

## Abstract

Hepatitis B virus (HBV) contains a 3.2 kb DNA genome and causes acute and chronic hepatitis. HBV infection is a global health problem, with 350 million chronically infected people at increased risk of developing liver disease and hepatocellular carcinoma (HCC). Methylation of HBV DNA in a CpG context (5mCpG) can alter the expression patterns of viral genes related to infection and cellular transformation. Moreover, it may also provide clues as to why certain infections are cleared or persist with or without progression to cancer. The detection of 5mCpG often requires techniques that damage DNA or introduce bias through a myriad of limitations. Therefore, we developed a method for the detection of 5mCpG on the HBV genome that does not rely on bisulfite conversion or PCR. With Cas9-guided RNPs to specifically target the HBV genome, we enriched in HBV DNA from primary human hepatocytes (PHHs) infected with different HBV genotypes, as well as enriching in HBV from infected patient liver tissue, followed by sequencing with Oxford Nanopore Technologies MinION. Detection of 5mCpG by nanopore sequencing was benchmarked with bisulfite-quantitative methyl-specific qPCR (BS-qMSP). The 5mCpG levels in HBV determined by BS-qMSP and nanopore sequencing were highly correlated. Our nanopore sequencing approach achieved a coverage of ~2000× of HBV depending on infection efficiency, sufficient coverage to perform a *de novo* assembly and detect small fluctuations in HBV methylation, providing the first *de novo* assembly of native HBV DNA, as well as the first landscape of 5mCpG from native HBV sequences. Moreover, by capturing entire HBV genomes, we explored the epigenetic heterogeneity of HBV in infected patients and identified four epigenetically distinct clusters based on methylation profiles. This method is a novel approach that enables the enrichment of viral DNA in a mixture of nucleic acid material from different species and will serve as a valuable tool for infectious disease monitoring.

## Data Summary

1. All raw and analysed sequencing data have been made publicly available with GEO accession number: GSE162518 (https://www.ncbi.nlm.nih.gov/geo/query/acc.cgi?acc=GSE162518).
2. Code for the methods used for performing *de novo* assembly, calling methylation and mapping epigenetic heterogeneity has been made available in GitHub: https://github.com/ChloeDG/CGpipe.
3. Genomes generated have been deposited in GenBank: accession number MW784518.
4. Detailed protocols developed for validating methylation with BS-qMSP have been made available on protocols.io: DOI: 10.17504/protocols.io.52bg8an (https://www.protocols.io/view/quantitative-analysis-of-methylation-and-hydroxyme-52bg8an).

**Impact Statement**

The efficient mapping of DNA methylation in viruses is paramount for understanding viral gene regulation and provides key information for the development of new anti-viral treatments for elimination or suppression. In this work we present a new method and approach to capture entire hepatitis B virus (HBV) genomes in a mixture of viral and host DNA using long-read sequencing. Using this technique we can study the genotype and DNA methylation patterns, in addition to distinguishing the different forms of HBV (rcDNA and cccDNA) at single-molecule resolution. This method can be adapted to capture and sequence other DNA viruses and represents an important advance in mapping methylation in viral genomes, as the first technique that does not rely on a reference genome, PCR amplification or bisulfite conversion.

## Introduction

Hepatitis B virus (HBV) infection is divided into five clinical categories: asymptomatic, acute, chronic, fulminant and occult. Occult HBV infection has been defined as the ‘presence of HBV viral DNA in the liver (with or without detectable HBV DNA in serum) of HBsAg-negative individuals tested with the currently available serum assays’ [1, 2]. Although the mechanism is not well understood, occult HBV infection is associated with liver pathogenesis, significantly increasing the risk for liver cirrhosis and hepatocellular carcinoma (HCC) [3–6]. In addition, the overall degree of viral replication has been strongly linked to carcinogenesis [7]. Therefore, understanding factors that regulate HBV replication may provide insights into occult infection and prevention of HCC.

HBV has a particular replication cycle that involves protein-primed reverse transcription of an RNA intermediate called pregenomic RNA (pgRNA) occurring in the nucleocapsid [8]. Upon entry into hepatocytes, viral genomic DNA in the nucleocapsid is in the form of 3.2 kb partially double-stranded DNA, known as relaxed circular DNA (rcDNA) (9). The rcDNA is then transported to the nucleus and converted into covalently closed circular DNA (cccDNA) by an ill-defined mechanism. The HBV polymerase, responsible for reverse transcription, is encoded within the viral genome. The HBV polymerase lacks proofreading activity, which results in a high amount of variability. Several HBV genotypes have been characterized, named A to J, along with nearly 40 sub-genotypes that have varying characteristics and clinical implications. These different HBV genotypes can vary in their DNA sequence by up to 7.5 % [10]. While our understanding of HBV DNA regulation is still limited, there is some evidence that cccDNA could be regulated epigenetically (reviewed in [11]).

Recent studies have identified epigenetic modifications of HBV DNA, including methylated cytosines (5mC), as a novel mechanism for the control of viral gene expression [12]. However, the current methods to detect modified bases have a number of limitations. Bisulfite modification has been considered to be the gold standard for the detection of 5mC for over a decade. This technique converts unmodified cytosines into uracil, after which 5mC levels are deduced by difference [13]. This technique leads to extensive DNA damage and introduces bias through incomplete conversion. Moreover, it is not able to distinguish the difference between 5mC and other modified bases that can occur in the same location (e.g. 5hmC). Thus, whilst this technique has been incredibly useful, there is a demand for the development of more direct measures of 5mC. Lastly, current sequencing methods are not able to easily distinguish between different forms of HBV (rcDNA verses cccDNA) or identify episomal vs integrated HBV DNA.

Nanopore sequencing is a unique, scalable technology that enables direct, real-time analysis of long DNA or RNA fragments [14]. It works by monitoring changes to an electrical current as nucleic acids are passed through a protein nanopore. The resulting signal is decoded to provide the specific DNA or RNA sequence. Moreover, this technology allows for the simultaneous detection of the nucleotide sequence as well as DNA and RNA base modifications on native templates [15]; hence, removing introduced bias from sodium bisulfite treatment and PCR amplification. However, enrichment of the target loci or species prior to sequencing is still necessary. Traditionally, this would be done by PCR amplification, but this would lead to a loss of modified bases such as 5mC. Thus, the development of enrichment techniques that do not degrade DNA or result in a loss of target bases is needed in order to fully benefit from Nanopore’s ability to delineate 5mC levels on native DNA. Nanopore is also capable of sequencing long reads, and can potentially capture the whole HBV genome in single reads and thus provide the sequence information for single HBV molecules. Integration of the entire HBV genome has not been reported, hence sequencing HBV with Nanopore has the potential to distinguish between episomal and integrated HBV DNA. Furthermore, sequencing single HBV molecules could also identify the ‘gap’ in rcDNA, allowing discrimination between rcDNA and cccDNA forms of HBV.

In this work we developed a translatable method for the enrichment, sequencing, *de novo* assembly and detection of modified bases in the HBV genome, determining the methylation landscape of different HBV genotypes *in vitro* as well as in patient liver tissue. We ascertained the HBV 5mCpG levels for the first time on directly sequenced native HBV DNA. These methods represent a valuable and highly novel tool for the detection of modified bases on viral genomes.

## Results

### Enrichment

**Fig. 1.**
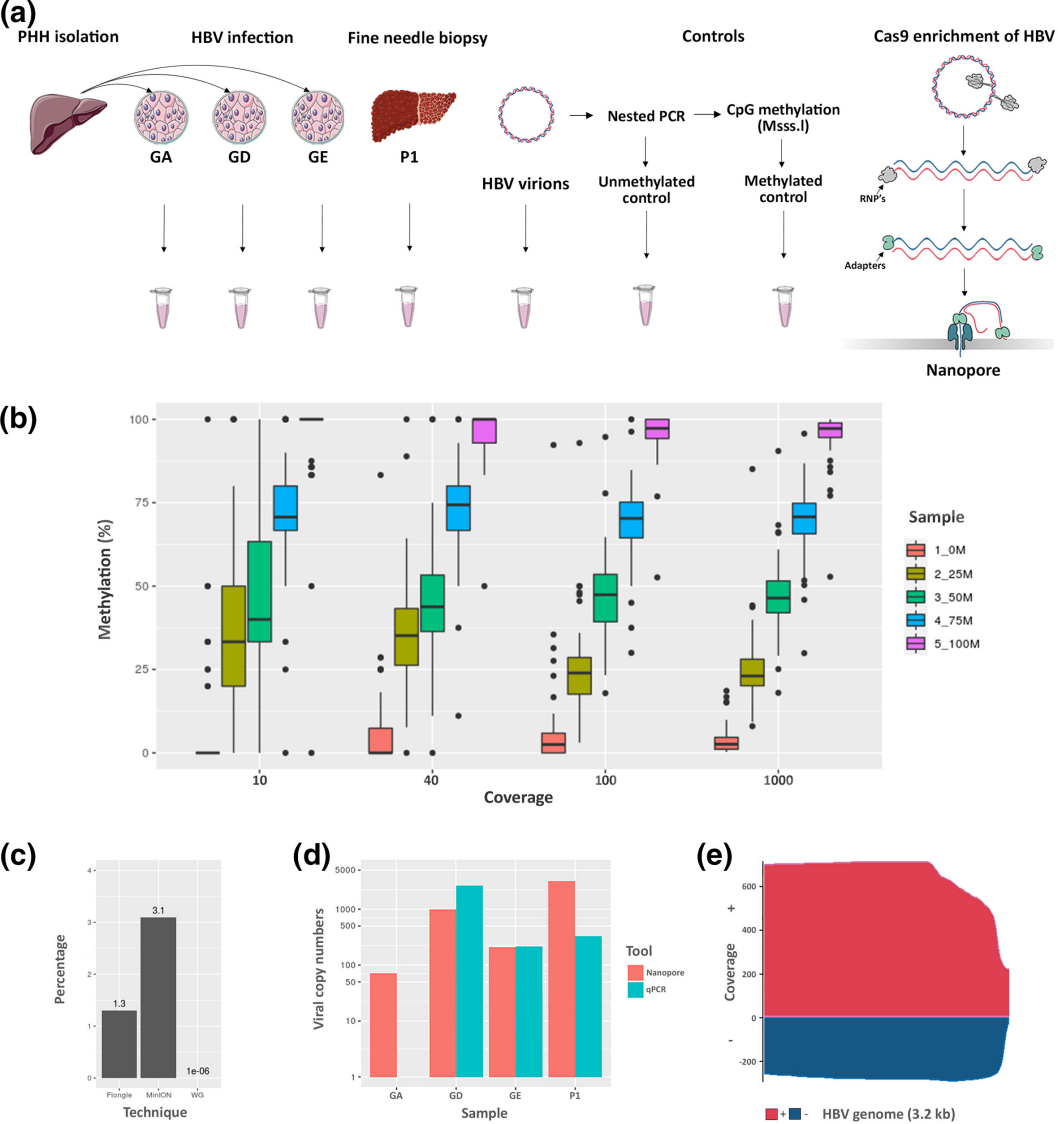
Enrichment and sequencing of HBV for the detection of methylation with Nanopore. (**a**) Overview of sampling and Cas9-targeted sequencing protocol adapted for circular viral genomes. Briefly, all available DNA ends were dephosphorylated prior to liberation of target sites by cutting with Cas9-guided RNPs (grey subunit). The circular viral genome was then linearized and prepared for the ligation of adapters and motor proteins (green subunit). Libraries were then loaded onto a MinION to be sequenced with nanopores. (**b**) Calculation of optimal coverage for HBV methylation detection. Briefly, reads from sequencing positive and negative controls for HBV methylation were pooled to achieve 10–1000× coverage and 0, 25, 50, 75 or 100 % methylation (0M, 25M, 50M 75M and 100M refer to the percentage methylation of each sample). (**c**) Yield of HBV aligned reads (percentage of total reads) using the Cas9 guide capture of HBV coupled with sequencing on MinION (MinION) and Flongle (Flongle) and HBV reads obtained with whole-genome nanopore sequencing on MinION (WG). (**d)** Sequencing depth achieved with Nanopore (reads aligning to HBV genome) compared to total HBV detected by qPCR (copies of HBV detected by qPCR). (**e**) Coverage of HBV genome from patient tissue (**p1**) after enrichment via Cas9 sequencing technique with MinION flow cell. *x*-axis, HBV genome length (3.2 kb); pink, positive-strand HBV; blue, negative-strand HBV.

Performing whole-genome sequencing of HBV infected cells without any type of enrichment achieves an extremely low sequencing depth with any sequencing platform. We performed whole-genome sequencing of HBV-infected primary human hepatocytes (PHHs) with Oxford Nanopore Technologies MinION and the percentage yield of HBV aligned reads was 0.0001 % (Fig. 1c). This was due to the size of the HBV genome compared to the contaminating host (3.2 kb vs 3.2 Gb, HBV=0.000001 %), and thus enrichment of HBV was necessary. We utilized Cas9-guided ribonucleoproteins (RNPs) to linearize and enrich in HBV DNA from the total DNA extracts. This method has been described previously for enrichment in target loci in the human genome [16], and to our knowledge, the present study provides the first evidence that this approach can be used to sequence viral or circular DNA (Fig. 1a), allowing enrichment without PCR amplification, and thus sequencing of native DNA while taking full advantage of the ability of nanopores to detect modified bases. Briefly, the starting material consisted of positive and negative controls for HBV methylation, total DNA extracted from PHHs infected with different genotypes (GA, GD or GE) or DNA from fine needle liver biopsies of chronic HBV-infected patients (P1) (Fig. 1a). Available DNA ends were blocked by dephosphorylation with calf intestinal phosphatase; this step is essential to prevent the nuclear DNA from being available for the ligation of adapters in later steps. We then used two single guide RNAs (sgRNAs) in a highly conserved region to target the HBV genome, one for the positive strand and an additional guide for the negative strand (Fig. 1a). The use of two gRNAs leading the positive and negative strands was critical, since the RNP complex remains attached to the strand where it makes the cut, making the other strand available for the ligation of adapters, allowing the sequencing of both the positive and negative strands. After the adapters and motor proteins were ligated to the newly available DNA ends, the libraries were loaded onto the MinION device and DNA sequencing occurred with 5′ to 3′ directionality on a MinION R9.4.1 flow cell.

### Yield and coverage

In order to determine the necessary sequencing depth to detect differential HBV DNA methylation, we combined reads from the positive and negative controls for methylation to obtain specific coverage (10, 40, 100 and 1000×) and percentages of methylation (0, 25, 50, 75, 100 %). We then determined methylation using Nanopolish and the minimum coverage was defined when a significant difference was detectable in each group of percentage methylation (Fig. 1b) (full list of *P*-values in Table S1, available in the online version of this article). Interestingly, even at the lowest level of coverage (10×) we observed a clear difference between the 0–75–100 % methylation levels (*P*<0.05). However, distinguishing between 25 and 50 % was not possible at this level of coverage. At a coverage of 40×, a significant difference in 5mCpG levels between each percentage methylation group was observed, and as such, 40× was identified as the minimum sequencing depth required for detection of HBV methylation.

Starting with DNA extracted from HBV virions, HBV-infected PHHs or infected patient tissue, we utilized our Cas9 enrichment protocol to linearize and sequence the HBV genome. The total yield ranged between 150-200000 K reads collected in the range of 1–2 Gb of DNA. Raw reads were basecalled with Guppy (version 4.0) and a draft assembly was generated with Canu (version 2.1) and polished with Medaka (version 1.0.3). The resulting consensus sequence was used to align the basecalled reads and calculate HBV genome coverage (Fig. 1e) and enrichment.

We obtained a clear enrichment of HBV with up to 3000× coverage for certain HBV genotypes, which was comparable with total HBV detected in the same samples by qPCR; variability in the sequencing depth can be explained by infection efficiency (Fig. 1d). On-target reads from MinION sequencing ranged from 3–10 % (Fig. 1c), representing a clear enrichment for this particular technique considering the very low levels of HBV detected with whole-genome sequencing. The Cas9 enrichment was able to capture entire HBV genomes along with shorter HBV reads that represent the partially reverse-transcribed positive strand (Fig. S1c). Significantly, the gap present in the positive strand of rcDNA that was identified in this analysis was most pronounced in HBV virions (Fig. S1c, e–f); a ~300 bp region starting at the end of the gene encoding for HBV polymerase. Surprisingly, we observed completed positive strands in HBV virions (<0.01 %), likely arising due to contamination of cccDNA from the viral generation process or an artefact of HBV replication.

Levels of infection of the PHHs used in this experiment were evaluated by qPCR, which established a high infection level (Figs 1d and S1g, h). Given the high copy numbers of viral DNA, and thus replicative intermediates, it was expected to find this range in the length of the gap present in the positive strand of rcDNA, with a clear tapering of read length; this could be at least partially due to the different stages in the transcription of HBV DNA (Fig. S1c). Interestingly, we observed a difference in the coverage of positive and negative strands, likely due to differences in the efficacy of the sgRNAs. Despite the differences in frequency, we were clearly able to sequence both strands of native HBV with the Cas9 enrichment technique at a coverage considered to be highly satisfactory for the identification of HBV methylation.

### Nanopore Flongles capable of sequencing native HBV in a mixture of viral and host DNA

In order to develop a more affordable and translatable method for the identification of HBV DNA in a mixture of viral and host DNA, we decided to test our method on the smaller nanopore flow cells, ‘Flongles’. Using these smaller flow cells allowed us to start with less DNA (0.5–1 μg). This is an important consideration for clinical translation of this method, since obtaining large quantities of DNA from tissue/liquid biopsies is not always feasible. After 4 h of sequencing we generated 367 reads totalling 1.3 MB of sequencing data. One hundred reads passed quality control (QC), and 1.3 % of these aligned to the HBV genome (Fig. 1c). However, we must note that the reads obtained from the Flongle were of a much lower quality than those sequenced with the MinION flow cells (Fig. S2d). While this was likely due to the lower molarity of the DNA available for sequencing causing an increase in the speed at which DNA passes through the nanopores, causing a drop in translocation speed, it is nonetheless an important consideration for the potential applications of this technique. As expected, by using Flongles and starting with a lower quantity of starting material, we obtained lower sequencing depth of the HBV than by using the larger MinION flow cells (Fig. 1c). Importantly, the same amount of starting material would have resulted in far greater coverage on the larger MinION flow cell. Nevertheless, our data suggest that our method can be applied to enrich HBV DNA in samples for sequencing with nanopore ‘Flongles’ to obtain rapid detection of HBV in laboratory infection models.

### Nanopore sequencing detects HBV 5mCpG

We next sought to determine the validity of using nanopores to detect modified bases in the HBV genome. Beta values for each CpG site were calculated by Nanopolish [17]. Our minimum coverage calculations (Fig. 1b) indicated that a minimum of 40× was required to detect a significant difference between each methylation interval (Table S1). However, at a coverage of 10× we were able to identify differences between 0 and 25% methylation, as well as 75 and 100% methylation. Thus a coverage of 10× for certain applications could be considered sufficient as is also the case for other techniques using short-read sequencing [18] and long-read sequencing [19].

**Fig. 2.**
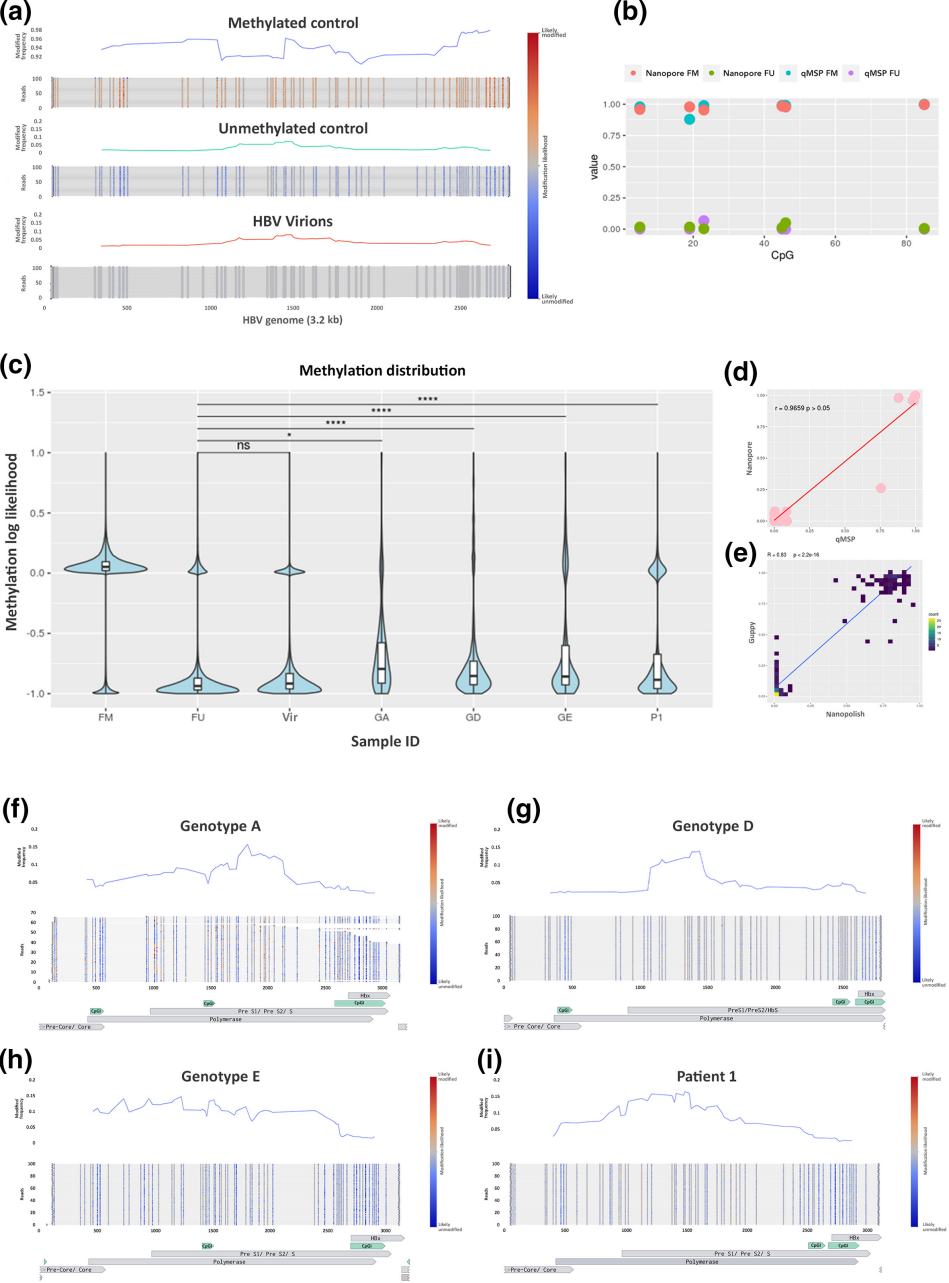
HBV methylation levels in HBV. (**a**) Average methylation of HBV controls and single-molecule visualization of first 100 reads using Methplotlib and (**b**) 5mC levels of 6 CpG sites detected using two techniques, nanopore sequencing and qMSP. (**c**) Distribution of 5mC in HBV from PHHs infected with different genotypes (GA, GD and GE) and isolated from patient tissue (**p1**) and fully methylated (FM) and unmethylated (FU). (**d**) Correlation of 5mC levels obtained with qMSP with Nanopore, samples+controls. (**e**) Correlation of 5mC levels detected with different methylation callers, Nanopolish and Guppy. (**f–i**) Methylated frequency and single-molecule visualization of HBV from infected PHHs (F, genotype A; G, genotype D; H, genotype E) and infected patient tissue (I=P1) (blue, unmethylted CpG site; red, methylated CpG site; CpGI, CpG Islands detected with Meth primer).

Nanopore sequencing directly evaluates methylation patterns on native DNA strands, and as such, we were able to observe long-range methylation information on each HBV genome, which was plotted with Methplotlib [20] (Fig. 2a, f–i). As anticipated, very low levels of methylation were observed in the negative control, with some residual methylation and background noise detected (Fig. 2a). Interestingly, we identified some single reads that were methylated in the negative control, likely as a result of the presence of residual unamplified DNA; we determined that the low levels of methylation observed were partially attributable to this contaminating starting material. However, we also identified random methylated CpG sites throughout reads in the fully unmethylated control. These could also have been due to methylation calling errors and can therefore be considered to be background noise for the technique. In the positive control we detected high levels of DNA methylation (Fig. 2a). However, there was variability in the average levels of 5mCpG. After visualizing the methylation of the single molecules, we identified a number of reads that were not fully methylated, which was likely contributing to the lower average levels of 5mCpG at certain loci. These unmethylated reads are likely attributable to the efficiency of the methyltransferase in the preparation step of the fully methylated control. However, in a similar way to the unmethylated control, there were some random CpG sites throughout the data that were not methylated in the positive control, which is more likely attributable to methylation calling errors. In order to address the issue of bias introduced through methylation callers, in addition to calling 5mCpG with Nanopolish, we validated our findings with an additional methylation caller, Guppy. Spearman’s correlation of the methylation levels obtained for the fully methylated and unmethylated HBV controls with Nanopolish and Guppy+Medaka indicated a highly significant correlation (*R*=0.83, *P*<2.2^−16^) (Fig. 2e).

To validate the findings obtained with Nanopore, we developed a bisulfite quantitative methyl-specific qPCR assay (BS-qMSP). Briefly, DNA from controls and genotype D samples were bisulfite-converted, followed by methyl-specific qPCR, with methyl-specific primers designed for six CpG sites in the HBV genome. Beta values were calculated by comparing the percentage (%) of methylated DNA to the total (unmethylated+methylated DNA) (Fig. 2b). As expected, beta values were comparable for all CpG sites in the methylated and unmethylated controls and samples, (Fig. 2d). The clear extremes observed in the different controls (Fig. 2a) (>~90 % methylation in FM, <~10 % methylation in the FU) validated in multiple methylation callers (Fig. 2e), and with an additional technique (Fig. 2b, d), indicate the efficacy of Nanopore technology for the detection of 5mCpG on HBV DNA. We were therefore highly confident in this tool for the detection of 5mCpG levels on the HBV genome.

### Absence of DNA methylation observed in HBV virions

After identifying the background noise levels of 5mCpG identified in synthetically fully methylated and unmethylated HBV DNA, we sought to determine the 5mCpG levels in an expected biologically negative control, HBV virions. HBV DNA is reverse-transcribed after being packaged in the viral capsid in the cytosol of the infected cells, and is thus out of touch with DNA methyltransferase enzymes, and, as a result, likely not methylated [8]. Overall, very low levels of 5mCpG were identified in the HBV virions, comparable to the amplified HBV control (Fig. 2a). These levels were not higher than those detected in the unmethylated HBV control (Fig. 2c) and, as such, were likely a result of methylation calling errors or potentially some contaminating DNA from dead cells also collected during the HBV viral particle purification process. Regardless, the levels were low (<10 %). Taken together with the methylation levels in the amplified, unmethylated HBV control, these levels of 5mCpG indicate the background noise levels of the detection method. Moreover, these data continued to increase our confidence in nanopore sequencing to accurately detect 5mCpG in the HBV genome.

### Basal levels of HBV 5mCpG in infected PHHs with different genotypes

In order to determine the basal levels of 5mCpG in HBV infection models *in vitro*, we used PHHs, which are the gold standard model for HBV infection *in vitro*. PHHs were infected with a viral inoculum of HBV genotypes A, D and E (GA, GD and GE) and collected nuclear DNA from cells 6 days post-infection. Infection efficiency was determined by HBsAg and HBeAg expression by ELISA as well as total HBV DNA in the supernatant by qPCR every 3 days. We observed similar infection rates for HBV genotypes D and E, but for genotype A all parameters were negligible, indicating lower infection rates (Fig. S1g, h). Despite the negligible levels detected for the infection efficacy of HBV genotype A, our Cas9 enrichment technique was able to enrich and sequence HBV genotype A with ~80× coverage (Fig. 1d). In addition, our Cas9 technique was highly effective at enriching HBV of all genotypes tested at sufficient coverage to evaluate methylation levels.

Detected 5mCpG levels were comparable across the different HBV genotypes (Fig. 2f–h). We observed low levels of 5mCpG across the three genotypes tested, with HBV genotype E having the highest basal levels across the genome (Fig. 2c). While we did not identify any differentially methylated loci when comparing the different genotypes, the technique developed was clearly able to enrich and sequence all genotypes tested. Moreover, the distribution of methylation for each of the different genotypes was significantly different from that of both of the negative controls (Fig. 2c) and from each other’s. Interestingly, we identified an enrichment of 5mC in the *preS1/preS2* region for genotypes A and D, which could have implications for HBs expression. These differences in 5mC distribution, taken together with the different pattern of methylation frequency (Fig. 2f–h), indicate that HBV genotypes exhibit genotype-specific 5mC landscapes. Thus, these data further add to the efficacy of the Cas9 sequencing technique for the enrichment and delineation of HBV methylation in a laboratory setting.

### Enrichment and sequencing of HBV in infected patients identifies epigenetic heterogeneity

**Fig. 3.**
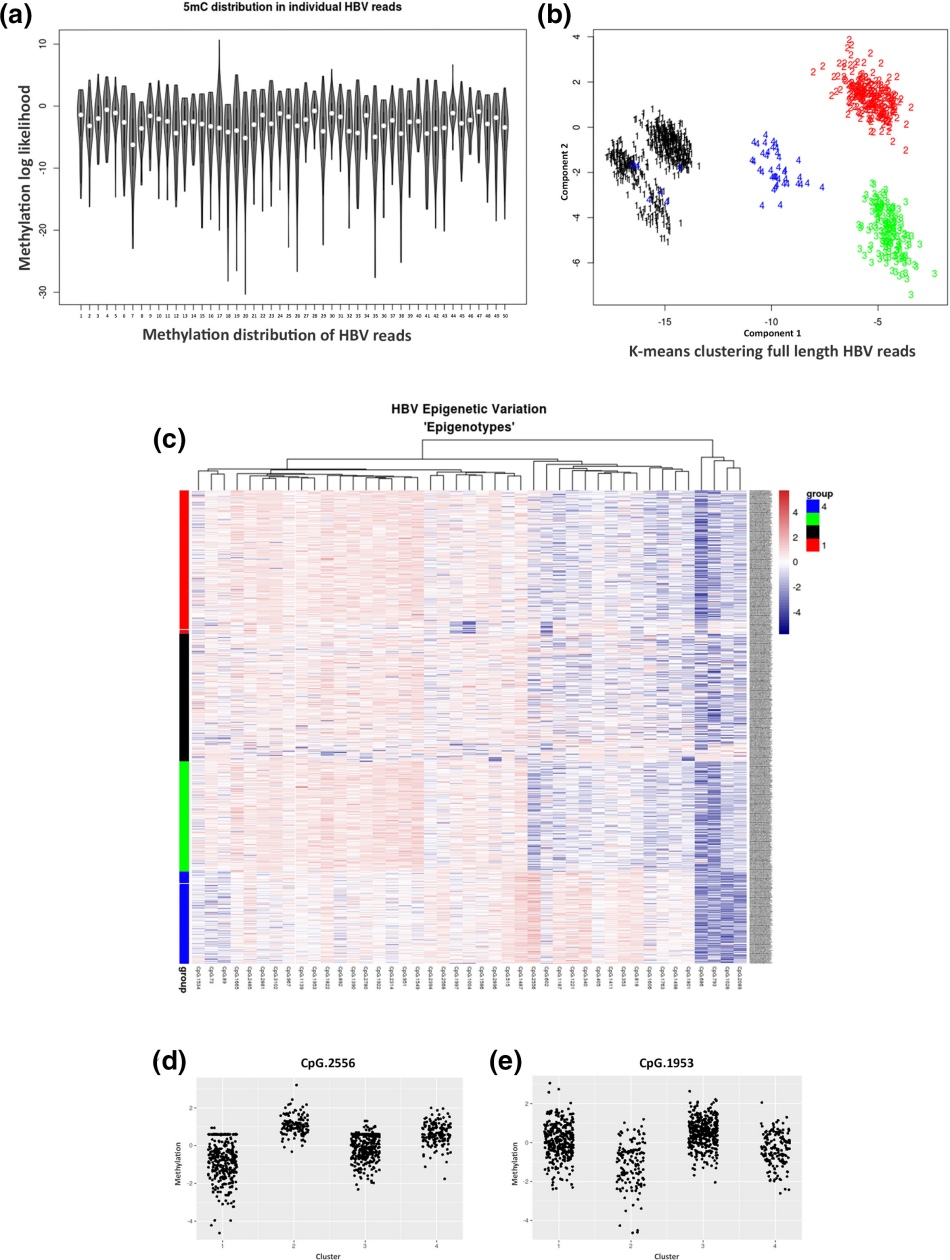
Epigenetic heterogeneity in HBV from infected patients. (**a**) Variability of 5mC levels in a random selection of single HBV molecules. (**b**) K-means clustering of HBV molecules. **(c**) Heatmap clustering of single HBV molecules. (d, e) Methylation distribution observed at CpG sites 1953 and 2556 in each cluster or ‘epigenotype’. Cluster numbers correspond to the same groups in Fig. 2b, c.

In order to test the efficacy of our technique and its potential for translational research in clinics, we tested Cas9 enrichment and sequencing of HBV DNA from patient tissue. We took advantage of samples collected as part of the PROLIFICA study [21] using 900 ng of DNA obtained from an HBV-positive patient’s liver biopsy. All viral parameters were assessed, including pgRNA and cccDNA quantification (Table S3). By using our Cas9 sequencing approach, we were able to enrich in enough HBV-specific reads to perform a *de novo* assembly. The HBV genotype was determined by the jumping profile hidden Markov model (jpHMM) and identified as genotype A (Fig. S1b) and, after alignment, identified over 1000× coverage of this HBV genome after filtering out reads <2.5 kb (Figs 1e and S1d). The methylation of CpG sites was determined and the average 5mCpG levels were calculated (Fig. 2i). Interestingly, certain CpG sites were around 50 % methylated across the HBV genome. Visualization of single HBV DNA molecules revealed heterogeneity in the methylation levels, indicating the potential existence of differentially methylated HBV populations within the patient. In order to further explore these data, we evaluated the distribution of HBV methylation levels in the patient sample. We observed a significant difference in the distribution of 5mCpG compared to that for both the fully methylated and fully unmethylated controls (Fig. 2c). Visualization of the 5mCpG distribution of individual reads (Fig. 3a) revealed heterogeneity of HBV molecules. Unsupervised hierarchical clustering of whole HBV molecules identified four distinct clusters (Fig. 3b, c), suggesting that within the one patient up to four epigenetically distinct HBV phenotypes existed. Whilst the differences between clusters were not identified as a particular region, there were certainly several CpGs that displayed large differences between groups. In particular, CpG in the *preS1/preS2* s region displayed large differences between clusters (Fig. 3d, e). While these data need to be confirmed with additional patients to draw broader conclusions, they are not entirely surprising; HBV replication intermediates and virions were not methylated, and since the methylation levels observed are based on full-length HBV genomes, it can be deduced that the observed 5mCpG is based on the presence of cccDNA. An understanding of the epigenetic landscape of different HBV DNA populations is important when considering HBV regulation and occult infection. Taken together, these data highlight the potential of this technique in the enrichment and sequencing of HBV as well as analysis of HBV DNA methylation in clinical settings.

## Discussion

HBV DNA methylation in infected cells can alter the expression patterns of viral genes related to infection and cellular transformation [22, 23] and may also aid in understanding why certain infections are cleared or persist with or without progression to cancer [24]. Furthermore, the clear detection of viral methylation patterns could potentially serve as biomarkers for diseases that are currently lacking, including occult HBV infection [25]. However, the development of more sensitive high-throughput techniques translatable to the clinic is essential. The present study proposes a technique to enrich and sequence HBV without the need for bisulfite conversion or PCR. Using Cas9-guided RNPs coupled with nanopore sequencing, we were able to enrich and sequence HBV from both infected PHHs and patient liver tissue, achieving coverage of ~2000×, providing the first *de novo* assembly of native HBV DNA, as well as the first landscape of 5mCpG from native HBV sequences. Furthermore, by using long-read sequencing, we captured entire HBV genomes (3.2 kb) and identified several HBV epigenotypes in patient tissue.

The Cas9 enrichment technique was first designed to enrich in target regions in the human genome prior to sequencing [16]. We have adapted this method to enrich in viral DNA in a mixture of host DNA. The coverage obtained for the previous study was ~400× and used a triple cutting approach, whereby six or more sgRNAs were designed for each region of interest. By using just two sgRNAs targeting the HBV genome, we obtained much higher coverage than in the previous study and dramatically reduced the cost per sample. We attributed this to the smaller size of the HBV genome compared to the average size of the regions the authors were targeting (3.2 kb vs 20 kb) as well as the higher concentration of HBV genomes per cell. Thus, it is not entirely surprising that the coverage was improved by such a large factor (>25x).

The developed technique enriches for episomal HBV DNA and can also distinguish between the different HBV forms. We first blocked linear DNA ends by phosphorylation and then linearized HBV with Cas9-guided RNPs, and after sequencing we selected reads between 2.5 and 4 kb to perform a *de novo* assembly. In doing so, we were sure to remove any integrated HBV reads that were sequenced, increasing specificity for episomal HBV. Moreover, we identified the ‘gap’ in the positive strand of HBV. Therefore, during post-sequencing analysis, by only selecting HBV reads that covered the whole-genome, we identified those reads that corresponded to cccDNA positive strand. However, since there is no gap in the negative strand of HBV rcDNA, we cannot distinguish it from cccDNA in this way. Other techniques to study rcDNA and cccDNA are limited. Southern blot remains the gold standard for quantification of cccDNA, but it is time consuming and not practical for a large number of samples [26]. Other techniques rely on PCR but show limited specificity when an excess of incomplete HBV rcDNA is present. Furthermore, HBV is renowned for its genomic sequence variability, with different genotypes varying by as much as 7.5 % [10], which can causes difficulties for the design of primers. Our Cas9 enrichment protocol was capable of sequencing all HBV genotypes tested. This is likely due to the conservation of the region used for the guides designed to specifically target HBV DNA, which was not bound by polymerase kinetics. Our Cas9 technique was also capable of enriching in HBV from patient liver tissue, achieving over 2000× coverage of the HBV genome and thus demonstrating the potential of this technique for translation to clinical applications.

Current strategies for DNA methylation determination include bisulfite conversion followed by Sanger sequencing or methyl-specific PCR, enzyme digestion assays and single-molecule, real-time (SMRT) sequencing [18]. It is difficult to compare the efficiency of different techniques for detecting DNA methylation, since they are all valuable for different applications; despite this, thorough reviews and comparisons have been conducted, with the limitations of each platform being well described [18]. Our technique was capable of detecting 5mCpG in native HBV DNA from different genotypes *in vitro*, as well as in patient liver tissue. An advantage of our Cas9 enrichment technique compared to traditional bisulfite-based sequencing methods is the clear benefit of sequencing native DNA that does not require bisulfite conversion. Bisulfite conversion results in DNA degradation and, importantly, is not capable of distinguishing 5mC from other modified bases, such as 5hmC, whereas nanopore-based techniques are able to do so. In addition, with this nanopore technique simultaneous elucidation of DNA sequence and single-molecule methylation was obtained, while other techniques such as bisulfite conversion followed by methyl-specific PCR require additional experimental analysis to ascertain the sequence information. Furthermore, by sequencing full HBV reads we are able to identify the heterogeneity of HBV sequences, making it possible to identify certain reads that are displaying differential methylation patters within the sample population, another application that is not possible with existing techniques. The exception, of course, is SMRT sequencing with PacBio.

While the nanopore platform has clear benefits, there are also several limitations. Importantly, a large quantity of starting material is required for this technique (1–5 µg of DNA). While this is true on paper, we were able to achieve 2000× coverage of HBV with 900 ng of starting material (liver tissue from an HBV-infected patient). On average our patient liver tissue biopsies are <25 mg and, depending on the DNA extraction technique, we can obtain up to 1–2 µg of DNA. However, for other patient samples such as blood and plasma, it is simply not possible to obtain large quantities of DNA. As such, PCR and bisulfite-based methods must be preferred. Nevertheless, with the development of the smaller nanopore Flongle flow cells and the continually improving machine learning applications [27], it will soon be feasible to combine our technique with computational approaches to enrich in viral DNA from much lower quantities of starting material.

While we have focused on the application of this technique for detecting modified viral DNA bases, this tool can also be useful for other approaches, including the identification of mutations and tracking of viral evolution, and the investigation of transmission chains, such as were observed in the Ebola outbreak in West Africa [28] and more recently in the COVID-19 pandemic of 2020–2021 [29]. By sequencing native reads and performing a *de novo* assembly, bias introduced from PCR amplification is eliminated. However, nanopore sequencing is still considered to be error prone, with the raw read accuracy varying depending on several factors, such as flow cell type, starting material quality, basecaller, basecalling speed, polishing, etc. In optimal conditions, nanopore sequencing has a reported raw read accuracy of ~97 % using flow cells with the R9.4 pore chemistry, while the consensus accuracy and single-molecule consensus accuracy is usually ~99.98 % (Q37) [30]. In the present study, our *de novo* assembly of native HBV reads generated a consensus with per-base 99.9 % similarity to existing HBV genotype D references generated with Illumina sequencing (Fig. S1b), which is comparable to the benchmark values for the assembler used (Canu) [31, 32]. Accurate references are imperative to improve the accuracy of downstream analysis, such as methylation calling.

Furthermore, there is huge potential for nanopore technology and the identification of additional modified bases. Since only low levels were observed and only one patient biopsy was screened in the present study, we were not able to correlate methylation with functional outcomes such as expression of viral parameters. That being said, the study of epigenetics is still in its infancy. Recently we found that levels of 5hmC in gene bodies in differentiating hepatocytes is highly correlated with the expression of those genes [33]. Thus, there is still a lot more to discover regarding the function of modified bases. However, the first point of call is accurate detection, and more work is needed to develop machine learning tools to detect and benchmark additional modified bases in native nanopore data.

## Conclusions

We developed a sensitive and high-throughput method for the enrichment and nanopore sequencing of native HBV DNA from infected PHHs and patient liver tissue. This method is a novel approach that achieved a clear enrichment of viral DNA in a mixture of virus and host DNA without the need for PCR amplification. By sequencing native HBV DNA with Nanopore we were also able determine the DNA methylation landscape of HBV without the use of bisulfite conversion. Moreover, using the developed technique, we have provided the first *de novo* assembly of native HBV DNA, as well as the first landscape of 5mCpG from naive HBV DNA. More work is needed to test compatible machine learning enrichment techniques to further improve the minimum required concentration of starting material.

## Methods

### Cultivation of PHHs

Primary human hepatocytes (PHHs) were extracted and maintained as described previously [34]. Briefly, PHHs were prepared from surgical liver resections with a two-step collagenase perfusion.

### HBV cultivation and infections

HBV inocula was generated as described previously [34, 35]. PHHs were naturally infected with HBV genotypes A, D and E for 24 h (m.o.i. 100). A stable infection was achieved after 3 days, and cells and DNA were extracted after 6 days. Infection efficiency was determined by quantification of hepatitis B surface antigen (HBsAg) and hepatitis B e antigen (HBeAg) concentration in supernatant by ELISA and calculation of HBV copies µl^−1^ by qPCR as described previously [34].

### Patient liver tissue

Patient samples were collected as a part of the PROLIFICA study [21, 36]. DNA was extracted from snap-frozen human liver needle biopsies. Liver samples were first homogenized on ice using a TissueRuptor (Qiagen, Hilden, Germany) in homogenization buffer (Tris/HCl pH 8, 50 mM; EDTA 1 mM; NaCl 150 mM) and then processed for DNA extraction.

### DNA extraction

Cells or homogenized tissues were digested with proteinase K prior to DNA isolation using the MasterPure DNA Purification kit (Epicentre, Illumina, Madison, WI, USA) according to the manufacturer’s instructions.

### Quantification of total HBV-DNA, cccDNA and pregenomic(pg) RNA in liver sample

Quantification was performed using the QX200 Droplet Digital PCR System (BioRad, Hercules, CA, USA) with primers and fluorescence dual hybridization probes specific for total HBV DNA or cccDNA as described elsewhere [37, 38]. Before cccDNA amplification, DNA was treated with 10U of Plasmid-safe Dnase (Epicentre, Illumina) for 45 min at 37 °C following the latest update of an international working group on cccDNA standardization (39). Serial dilutions of a plasmid containing an HBV monomer (pHBV-EcoR1) served as quantification standard. To normalize the number of viral copies per cell content, the number of cellular genomes was determined using the b-globin genekit (Roche Diagnostics, Manheim, Germany). The patient sample was analysed independently in duplicate. The range of quantification was between 10^1^ and 10^7^ copies of HBV genome/well for both cccDNA and total HBV-DNA assays. For pgRNA detection, specific primers and the Taqman hybridization probe were used, as described elsewhere [37, 38]. The patient sample was analysed independently in duplicate and the relative amount of pgRNA was normalized over the expression of the housekeeping the gene GUSB (Hs99999908_m1, Thermo Fisher Scientific, Waltham, MA, USA).

### Laboratory assays

HBsAg was detected by chemiluminescent microparticle immunoassay (Architect, Abbott) in 2012–2013 [40]. HBV DNA levels were measured at the end of the Prolifica study in stored serum samples using in-house quantitative real-time PCR (detection limit: 50 IU ml^−1^), calibrated against an international standard [41].

### Fully unmethylated and fully methylated controls

HBV DNA was amplified by nested PCR as described previously to prepare a negative (fully unmethylated) control. After amplification, a positive control for methylation (fully methylated) was prepared by methylating CpG dinucleotides – by incubating 1 µg of DNA with S-adenosyl methionine (SAM) (32 µM) with CpG methyltransferase (M.SssI) (4–25 units) (New England BioLabs) at 37 °C for 1 h before heating to 65 °C for 20 min.

### Nanopore library prep and sequencing

DNA (0.5–3 µg) from each sample or control was enriched in HBV and linearized using Cas9 guide RNPs. TracrRNA and crRNA (5′AGCTTGGAGGCTTGAACAGT3′ and 5′TAAAGAATTTGGAGCTACTGTG3′) were purchased from Integrated DNA Technologies (IDT). Samples were barcoded and multiplexed using the Nanopore Ligation Sequencing kit (SQK-LSK109) and Native barcode expansion kit according to the manufacturer’s instructions (Oxford Nanopore Technology, Oxford, UK). Sequencing was conducted with a MinION sequencer on ONT 1D flow cells (FLO-min106) with protein pore R9.4 1D chemistry for 24 h or on a Flongle (FLO-106) for 4 h (Oxford Nanopore Technology, Oxford, UK). Reads were basecalled with Guppy (version 4.0). Short and long reads were filtered out (<2.5 kb and >4 kb, respectively) with NanoFilt and were not included in further analysis.

### *De novo* assembly

Basecalled fasQ files were used to assemble the HBV genome with canu [31], which was polished using Medaka; a tool to create a consensus sequence from nanopore sequencing data using neural networks applied from a pileup of individual sequencing reads against a draft assembly. Basecalled FastQs were then aligned to the generated consensus sequence using Minimap2 (42). Assembly was assessed by using blast [43], which returned a 99.9 % similarity score to existing HBV references and the jumping profile hidden Markov model (jpHMM) [44], which identified the genotypes correctly (Fig. S1b).

### Methylation calling

The methylation status of each CpG site on every read was determined using Nanopolish [17], as used recently elsewhere [19]. For validation, NA methylation was also called with Guppy (version 4.0). CpG Islands were predicted using MethPrimer.

### Bisulfite (BS) and quantitative methyl-specific PCR (qMSP)

BS and qMSP protocols were made available as detailed methods at protocols.io [45]. Primers were designed with MethPrimer and purchased from Thermo Fisher Scientific (Waltham, MA, USA); the sequences are available in Table S2.

### Statistics

Data processing and statistical analyses were performed in R bioconductor (version 4.0).

To detect 5mCpG differences, a Kruskal–Wallis test one-way analysis of variance with Bonferroni correction was applied to determine differences between percentage methylation (0, 25, 50, 75 and 100 %) at different coverages (10, 40, 100 and 1000×) and a Dunn’s test with Holm’s correction was used to identify differences between single-molecule methylation levels of different HBV genotypes and negative methylation controls (fully unmethylated and HBV virions) (significance value, *P*<0.01). Incomplete reads that were shorter than the full HBV genome were filtered out using Nanofilt (Fig. S1d). Clustering analysis was performed for each sample on the basis of reads, assuming that one read is derived from a single HBV genome. The goal was to identify subgroups of HBV genomes within each sample based on their methylation profile. To this end we estimated the optimal number of subgroups using the elbow method. This was followed by k-means clustering using using wald.d Euclidean-based distance as similarity measure.

### Converting reads for visualization

In order to take advantage of single-molecule sequencing with Nanopore, we converted the reads for visualization using Methplotlib [20].

### Data availability

All raw and processed sequencing datahave been made publicly available with GEO accession number GSE162518. Assembled HBV sequences have been made available in GenBank: accession number MW784518.

Supplementary material 1
