# Supplementary material for: Cas9-targeted nanopore sequencing reveals epigenetic heterogeneity after de novo assembly of native full-length hepatitis B virus genomes
Source: Microb Genom. 2021 May 18;7(5):000507. doi: 10.1099/mgen.0.000507 (PMC8209722; doi:10.1099/mgen.0.000507)
Supplement: Supplementary material 1 [file mgen-7-0507-s001.pdf]

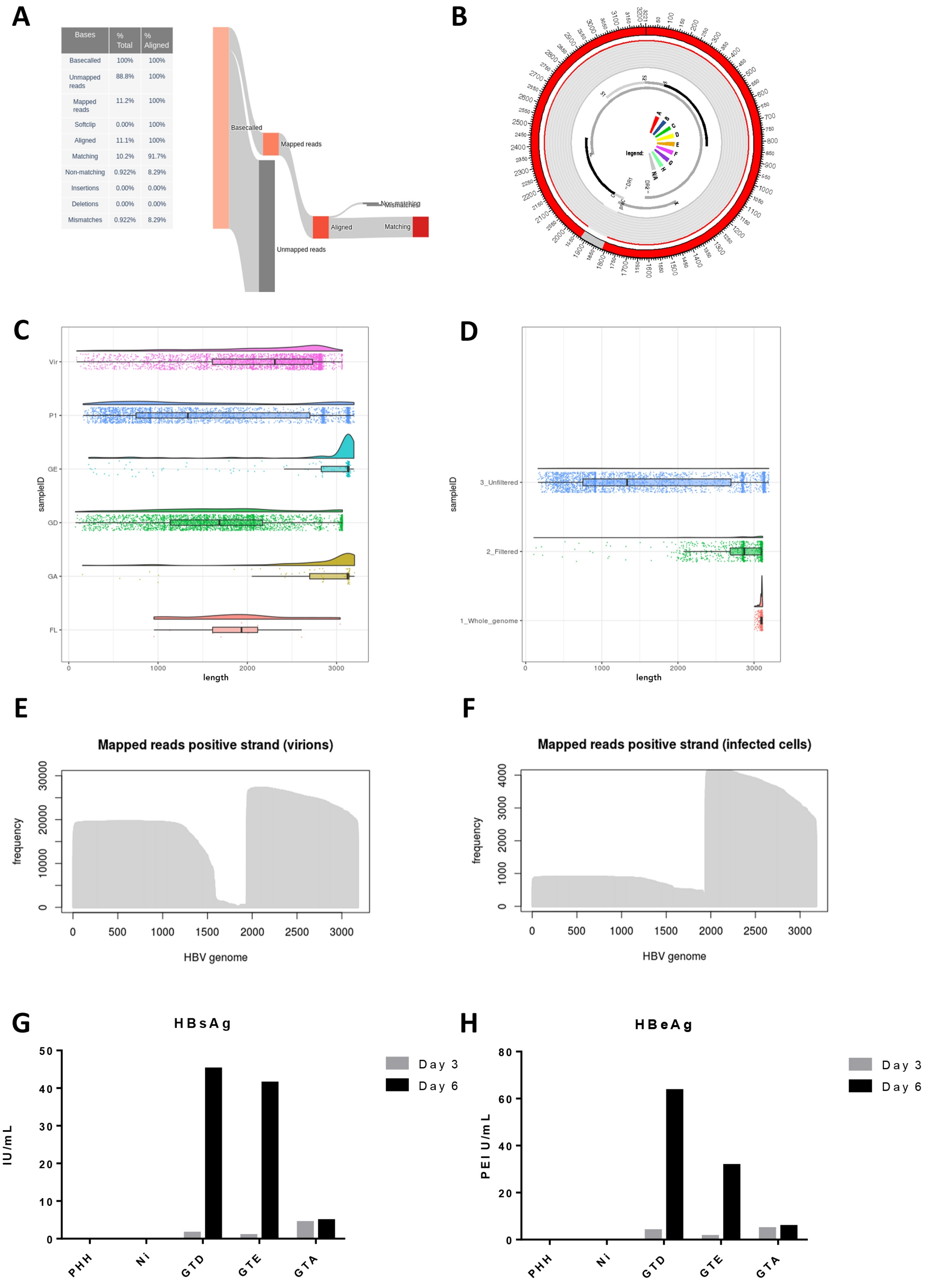
A
Bases
% Total
% Aligned
Basecalled 100% 100%
Unmapped reads 88.8% 100%
Mapped reads 11.2% 100%
Softclip 0.00% 100%
Aligned 11.1% 100%
Matching 10.2% 91.7%
Non-matching 0.922% 8.29%
Insertions 0.00% 0.00%
Deletions 0.00% 0.00%
Mismatches 0.922% 8.29%
Basecalled
Mapped reads
Unmapped reads
Aligned
Matching
Non-matching
Mismatches
B
legend:
C
Vir
P1
GE
GD
GA
FL
sampleID
length
D
3_Unfiltered
2_Filtered
1_Whole_genome
sampleID
length
E
Mapped reads positive strand (virions)
frequency
HBV genome
F
Mapped reads positive strand (infected cells)
frequency
HBV genome
G
HBsAg
IU/mL
Day 3
Day 6
PHH
Ni
GTD
GTE
GTA
H
HBeAg
PEIU/mL
Day 3
Day 6
PHH
Ni
GTD
GTE
GTA

Supplementary figure 1. **A** Yield for aligned reads calculated with pycoQC (representative sample). B: HBV genotype was determined by jumping profile Hidden Markov Model (jpHMM). C: Plot of total read length prior to filtering out short reads for all samples; HBV virions (Vir), patient enriched HBV (P1), HBV form infected PHH (GA, GD and GE) and HBV sequenced on Flongles (FL). D: plot depicting read length after each filtering stage, unfiltered = no filtering applied, filtered = removal of reads less than 2KB, whole genome = removal of all reads less than 3KB. E-F: Coverage of HBV virions and HBV isolated from infected PHH against adjusted reference genome starting at ECOR1 restriction site. G-H: Infection efficacy for HBV genotypes. PHH were infected with 100MOI of HBV geotype A, E or D. Serum levels of HBsAg, HBeAg were quantified by ELISA and HBV DNA was quantified by qPCR after 0, 3 and 6 days post infection. PHH = negative control, uninfected PHH only, Ni = negative control, uninfected PHH treated with PEG.

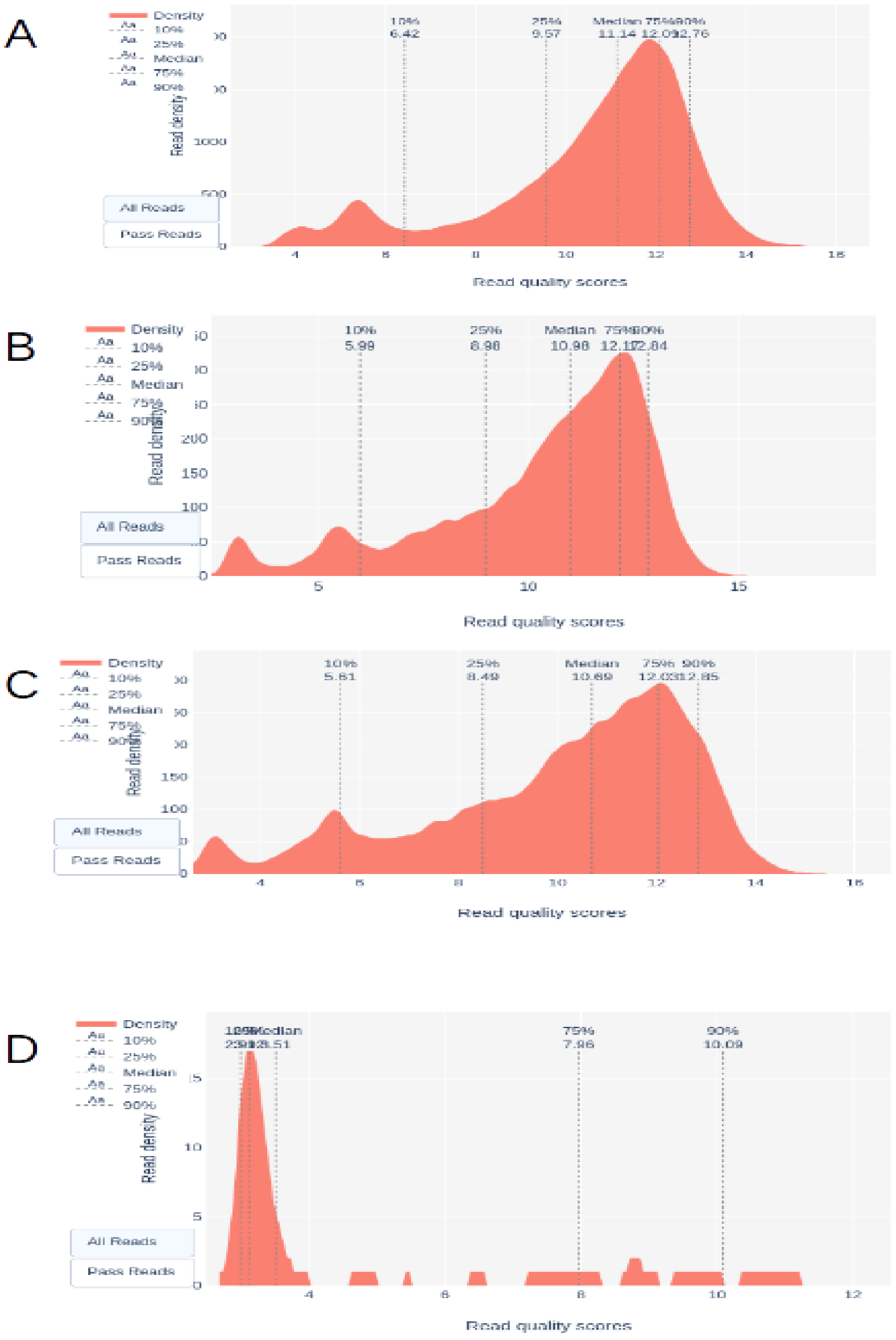

Supplementary figure 2. PHRED scores for nanopore runs. A: Genotype A sequenced on MinION, B: Genotype D sequenced on MinION, C: Genotype E sequenced on MinION, D: Genptype D sequenced on Flongle.

Supplementary table 1. Sequencing depth for effective calculation of HBV 5mCpG levels: p-value table corresponding to figure 1B.

coverage		Group 1	Group 2	statistic	p	padj	p.adj.significant
**10**	x	0% Meth	25% Meth	527.5	2.05E-12	8.2E-11	****
**10**	x	0% Meth	50% Meth	392	1.44E-14	5.76E-13	****
**10**	x	0% Meth	75% Meth	103	3.21E-20	1.284E-18	****
**10**	x	0% Meth	100% Meth	69	7.03E-23	2.812E-21	****
**10**	x	25% Meth	50% Meth	1391.5	0.043	1	ns
**10**	x	25% Meth	75% Meth	485.5	4.59E-12	1.836E-10	****
**10**	x	25% Meth	100% Meth	168	2.41E-19	9.64E-18	****
**10**	x	50% Meth	75% Meth	647.5	2.13E-09	8.52E-08	****
**10**	x	50% Meth	100% Meth	272	2.94E-17	1.176E-15	****
**10**	x	75% Meth	100% Meth	342	5.43E-16	2.172E-14	****
**40**	x	0% Meth	25% Meth	193	2.06E-17	8.24E-16	****
**40**	x	0% Meth	50% Meth	127.5	1.42E-18	5.68E-17	****
**40**	x	0% Meth	75% Meth	58	3.38E-20	1.352E-18	****
**40**	x	0% Meth	100% Meth	1.5	7.56E-22	3.024E-20	****
**40**	x	25% Meth	50% Meth	1148	0.00095	0.038	*
**40**	x	25% Meth	75% Meth	223	1.27E-16	5.08E-15	****
**40**	x	25% Meth	100% Meth	55.5	2.2E-20	8.8E-19	****
**40**	x	50% Meth	75% Meth	335	2.4E-14	9.6E-13	****
**40**	x	50% Meth	100% Meth	104	3.29E-19	1.316E-17	****
**40**	x	75% Meth	100% Meth	180	7.93E-18	3.172E-16	****
**100**	x	0% Meth	25% Meth	306.5	6.15E-15	2.46E-13	****
**100**	x	0% Meth	50% Meth	80	3.13E-19	1.252E-17	****
**100**	x	0% Meth	75% Meth	60	7.82E-20	3.128E-18	****
**100**	x	0% Meth	100% Meth	8	4.73E-21	1.892E-19	****
**100**	x	25% Meth	50% Meth	352.5	5.01E-14	2.004E-12	****
**100**	x	25% Meth	75% Meth	82	1.96E-19	7.84E-18	****
**100**	x	25% Meth	100% Meth	8	4.29E-21	1.716E-19	****
**100**	x	50% Meth	75% Meth	340	2.99E-14	1.196E-12	****
**100**	x	50% Meth	100% Meth	33.5	2.27E-20	9.08E-19	****
**100**	x	75% Meth	100% Meth	139.5	2.39E-18	9.56E-17	****
**1000**	x	0% Meth	25% Meth	41.5	4.12E-20	1.648E-18	****
**1000**	x	0% Meth	50% Meth	1	7.94E-21	3.176E-19	****
**1000**	x	0% Meth	75% Meth	0	5.18E-21	2.072E-19	****
**1000**	x	0% Meth	100% Meth	0	5.15E-21	2.06E-19	****
**1000**	x	25% Meth	50% Meth	232	3.03E-16	1.212E-14	****
**1000**	x	25% Meth	75% Meth	68	1.01E-19	4.04E-18	****
**1000**	x	25% Meth	100% Meth	4	4.31E-21	1.724E-19	****
**1000**	x	50% Meth	75% Meth	244	5.14E-16	2.056E-14	****
**1000**	x	50% Meth	100% Meth	18.5	1.31E-20	5.24E-19	****
**1000**	x	75% Meth	100% Meth	93.5	3.4E-19	1.36E-17	****

Supplementary table 2. Primer sequences for BS-qMSP of HBV.

NAME	SEQUENCE (5' > 3')<br>5-139 bases according to the synthesis scale
CpG1MF1	ATTTTTGGAAGAGAAATCGTTA
CpG1MR1	CGTCGTCTAACAACAATAATTTCC
CpG1UF1	ATTTTTGGAAGAGAAATTGT
CpG1UR1	AAAAAACCTACCTCATCATCT
CpG2MF1	TATATATTTTATGGAAGGCGGG
CpG2MR1	AACTAAATCCAACTAATAATCGAAA
CpG2UF1	ATATATTTTATGGAAGGTGGG
CpG2UR1	ACTCTAAAAACTAAATCCAACTAATAATCA
CpG3MF1	TAATAAGGTAGGAGTTGGAGTATTCG
CpG3MR1	ATTTCTCAAAAATAAAAACAACGAAA
CpG3UF1	AATAAGGTAGGAGTTGGAGTATTT
CpG3UR1	ATTTCTCAAAAATAAAAACAACAAAA
CpG4MF1	TGTTGTTGTATTAAATTTTCGGAC
CpG4MR1	AATAAACTAAACCAAAAAAAACGAA
CpG4UF1	TGTTGTTGTATTAAATTTTTGGATG
CpG4UR1	AATAAACTAAACCAAAAAAAACAAA
CpG5MF1	TTTCGTTTGTGTTTTTTTATTTGTC
CpG5MR1	AAAATATACCTCAAAATCGATCGTT
CpG5UF1	TTTGTTTGTGTTTTTTTATTTGTTG
CpG5UR1	AAAATATACCTCAAAATCAATCATT

Supplementary table 3. Viral parameters from HBV infected patient.

	Sample	Patient 1
**Biopsy tissue**	HBVtot/HBB	331.86
	cccDNA/HBB	2.13
	pgRNA/HBB	39.44
	pgRNA/cccDNA	20.38
**Serum**	Origin	blood donors
	Clinical Status	LC
	HBsAg	1
	Age	23
	Sex	1
	UrbanRural	R
	District	31
	OthersDistrict	1
	Ethnic	1
	PreS	0
	ORF size	1200
	AFB1 exposure	0
	R249S TP53 AF	2.009646E-05
	HBeAg	1
	VL	288720
	AST	45
	ALT	45
	Plats	217
	SRATE	80
	MSTIFF	10.2
	IQR	1.4
	IQRMED	14
